# Longitudinal fNIRS and EEG metrics of habituation and novelty detection are correlated in 1–18-month-old infants

**DOI:** 10.1016/j.neuroimage.2023.120153

**Published:** 2023-07-01

**Authors:** Laura Katus, Anna Blasi, Sam McCann, Luke Mason, Ebrima Mbye, Ebou Touray, Muhammed Ceesay, Michelle de Haan, Sophie E. Moore, Clare E. Elwell, Sarah Lloyd-Fox, the BRIGHT Study Team

**Affiliations:** a Institute for Lifecourse Development, School of Human Sciences, University of Greenwich, London, United Kingdom; b Centre for Family Research, University of Cambridge, Cambridge, United Kingdom; c Department of Medical Physics and Biomedical Engineering, University College London, United Kingdom; d Department of Women and Children's Health, Kings College London, United Kingdom; e Forensic and Neurodevelopmental Sciences, King's College London, United Kingdom; f MRC Unit The Gambia at the London School of Hygiene and Tropical Medicine, The Gambia; g Great Ormond Street Institute of Child Health, University College London, United Kingdom; h Great Ormond Street Hospital for Children NHS Foundation Trust, London, United Kingdom; i Department of Psychology, University of Cambridge, United Kingdom; j The BRIGHT Study team are (in alphabetical order): Lena Acolatse, Chiara Bulgarelli, Maria-Magdalena Crespo Llado, Momodou K. Darboe, Saikou Drammeh, Tijan Fadera, Giulia Ghillia, Buba Jobarteh, Marta Perapoch Amado, Andrew M. Prentice, Maria Rozhko, Mariama Saidykhan

**Keywords:** Habituation, Novelty detection, fNIRS, EEG, Neurodevelopment

## Abstract

**Introduction:**

Habituation and novelty detection are two fundamental and widely studied neurocognitive processes. Whilst neural responses to repetitive and novel sensory input have been well-documented across a range of neuroimaging modalities, it is not yet fully understood how well these different modalities are able to describe consistent neural response patterns. This is particularly true for infants and young children, as different assessment modalities might show differential sensitivity to underlying neural processes across age. Thus far, many neurodevelopmental studies are limited in either sample size, longitudinal scope or breadth of measures employed, impeding investigations of how well common developmental trends can be captured via different methods.

**Method:**

This study assessed habituation and novelty detection in *N* = 204 infants using EEG and fNIRS measured in two separate paradigms, but within the same study visit, at 1, 5 and 18 months of age in an infant cohort in rural Gambia. EEG was acquired during an auditory oddball paradigm during which infants were presented with Frequent, Infrequent and Trial Unique sounds. In the fNIRS paradigm, infants were familiarised to a sentence of infant-directed speech, novelty detection was assessed via a change in speaker. Indices for habituation and novelty detection were extracted for both EEG and NIRS

**Results:**

We found evidence for weak to medium positive correlations between responses on the fNIRS and the EEG paradigms for indices of both habituation and novelty detection at most age points. Habituation indices correlated across modalities at 1 month and 5 months but not 18 months of age, and novelty responses were significantly correlated at 5 months and 18 months, but not at 1 month. Infants who showed robust habituation responses also showed robust novelty responses across both assessment modalities.

**Discussion:**

This study is the first to examine concurrent correlations across two neuroimaging modalities across several longitudinal age points. Examining habituation and novelty detection, we show that despite the use of two different testing modalities, stimuli and timescale, it is possible to extract common neural metrics across a wide age range in infants. We suggest that these positive correlations might be strongest at times of greatest developmental change.

## Highlights

• We studied habituation and novelty detection via EEG and fNIRS at 1, 5 & 18 months.
• fNIRS and EEG responses were correlated for habituation (1&5 m) and novelty (5&18 m).
• Findings represent the first converging longitudinal infant fNIRS and EEG responses.
• Responses were associated across a wide age band despite using different stimuli.
• Cross-modality correlations may be strongest at times of great developmental change.

## 1 Introduction

Habituation and novelty detection are two core processes of neurodevelopment. The bias to prioritise stimuli that have not been previously encountered aids identification of meaningful signals, while not expending energy on recurrent but inconsequential stimuli (Eisenstein et al., 2001). Neural response suppression to repeated sensory input and increased responses to novel stimuli serves as an efficient means of directing attention and thus promote learning (Rovee-Collier and Cuevas, 2008). Being tightly bound to an organism's survival, habituation represents a low-level but crucial process, that has been studied across diverse species such as sea slugs (Castellucci et al., 1970), fruit flies (Duerr et al., 1982) as well as rats (Pilz et al., 1996) and primates (e.g., Baylis and Rolls, 1987, Miller et al., 1991).

Neural habituation and novelty detection responses have been well documented across the lifespan (for a review see Nordt et al., 2016), and across assessment modalities including functional magnetic resonance imaging (e.g., Bruckner et al., 1998), functional near infrared spectroscopy (fNIRS, Nakano et al., 2009), electroencephalography (EEG, e.g., Jacob et al., 2019) and magnetoencephalography (Ishai et al., 2006). Habituation and novelty detection provide good candidate processes for longitudinal studies from early infancy onwards: responses can be obtained in absence of overt behavioural responses from birth and can then be longitudinally studied using the same paradigms across wide age-ranges. However, there is a further need to validate indices across assessment modalities to examine whether the underlying cognitive constructs can be robustly assessed. This is particularly true for infants and children, as it is currently not known whether developmental effects are indicating the sensitivity of one modality at a specific age point or are capturing true underlying neurodevelopmental changes. The current study aims to begin to fill this gap, by longitudinally assessing neural specialisation associated with habituation and novelty detection at 1, 5 and 18 months of age in an infant cohort in The Gambia, West Africa. We examined whether indices from two neuroimaging modalities (fNIRS and EEG), capture similar developmental changes on group level, and whether infants’ individual responses in one modality correspond with their response in the other. Exploiting the strengths of each assessment modality, such an investigation could further inform our understanding of how brain function in response to habituation and novelty detection changes over time. Additionally, it could elucidate which functional changes are associated with a developmental change in the underlying neural circuitry associated with both processes.

### 1.1 Validation of assessment instruments and early neural measures

Even though both EEG and fNIRS have proven to be invaluable neurocognitive assessment tools during infancy and childhood, cross-modal studies assessing corresponding neural metrics across both measures are still rare. EEG provides a direct, highly temporally resolved measure of rapid changes in the functional activation of populations of neurons. Where a sufficiently high number of electrodes are being used, it is possible to also draw spatial inferences from the data (e.g., Xie et al., 2018). Data gathered in children and infants however is oftentimes restricted to low-density recordings, making spatial inferences based on EEG challenging. fNIRS on the other hand, provides a more spatially, but less temporally, resolved measure of the haemodynamic response occurring in relation to neuronal activation. It can therefore enable a better structure-to-function mapping, that is oftentimes not possible to achieve with infant EEG. Using the two methods in conjunction holds the potential to examine both the temporal changes in neuronal activation as it occurs and draw inferences about the spatial localisation of these processes, enabling a more complete picture of how activation in certain structures changes across development.

In clinical contexts, several studies have utilised concurrent fNIRS and EEG recordings to better understand haemodynamic response changes accompanying atypical electrophysiological activity (Bourel-Ponchel et al., 2017; Singh et al., 2014). However, adapting this clinical approach to a concurrent recording of paradigm- based designs poses challenges in terms of the timescale of the measured signal for EEG and fNIRS responses: while EEG and event related potentials (ERPs) allow for the presentation of a great number of stimuli presented approximately at a rate of one per 1–3 s, the haemodynamic response measured by fNIRS unfolds much more slowly and is usually captured during presentation of trials 5–20 s in length (Lloyd-Fox et al., 2010). This difference therefore necessitates the adaptation of stimulus timing to elicit meaningful responses in both modalities. Some important groundwork has been accomplished by Chen et al. (2015) in healthy adult participants: using a simultaneous set up of recording EEG and fNIRS, they demonstrated that the fNIRS signal showed regional specificity of activation over auditory and visual cortex, and that the degree of this regional specificity was associated with the magnitude of simultaneously recorded visually and auditory evoked potentials. Their study thus highlighted how using fNIRS and EEG concurrently can enable inferences on spatial (fNIRS) and functional (EEG) specificity of low-level neurosensory processes. Combined fNIRS/EEG approaches have also proven useful in studies on infants. For example, Telkemeyer et al. (2009) found differential effects for their EEG and fNIRS measures when presenting healthy newborns with auditory stimuli of varying durations. Differential haemodynamic responses over bilateral temporal cortex were measured via fNIRS, whereas no discriminatory pattern for stimulus duration could be found via the auditory evoked potentials. The authors concluded that this difference between the modalities might have been seen because auditory evoked potentials only reflect change detection during the initial presentation of a stimulus, and thus may not be a sensitive measure for condition differences such as the ones presented in this study. An examination of later EEG components might thus provide a better index of differential stimulus conditions. Obrig et al. (2017) demonstrated parallel effects in both modalities on an associative word-learning paradigm in 6-month-old infants, with both measures showing evidence for non-word learning over repeated sessions. However, a recent study in 18-month-old infants using a similar methodological set-up, Steber and Rossi (2020) found differential responses for linguistically legal vs. illegal pseudo-words in the infant's ERP, but no differential responses in their fNIRS signal. The authors suggest that these results could be associated with methodological limitations (specifically the stimulus timing required in parallel EEG/fNIRS recording) or be developmental in nature, with 18-month-old infants showing less robust neural responses to linguistic rule-violations than younger infants. These studies provide a crucial starting point in demonstrating cross-sectionally how EEG and fNIRS in conjunction can inform our understanding of early neurodevelopment. In summary, they highlight the need for further investigations of longitudinal changes in cross-modal associations over development. These would hold potential to understand whether certain developmental effects can be seen in different modalities at different ages, or whether they co-occur robustly across infancy. While this work on parallel recordings is currently underway, approaches whereby indices from each modality are measured sequentially one after the other can provide a first insight into common developmental trends as assessed by different measures. One limiting factor in this line of research is that the estimation of robust neurodevelopmental trajectories across more than one assessment modality is rarely feasible. Where such investigations are possible, sample sizes are often limited, which in context of higher rates of data rejection in infancy research, can pose a challenge when seeking to define longitudinal developmental trajectories. The current study assesses correlations between EEG and fNIRS responses measured sequentially within the same day within a longitudinal infant cohort at 1, 5 and 18 months of age. Hereby, we make use of a recent move towards studying neurodevelopment in large-scale infant cohorts in low-and middle-income countries, as this provides an ideal context to address questions regarding the robustness of different neurodevelopmental metrics across a wide developmental time window.

### 1.2 Large- scale global health studies provide framework for longitudinal, cross-modal investigations

Over recent years, an increasing number of projects have begun to examine neurodevelopment in low-and middle-income countries (Larson et al., 2019; Turesky et al., 2020; Wijeakumar et al., 2019; Xie et al., 2019). Neuroimaging represents a crucial tool in studying young infants from diverse cultural backgrounds: paradigms can be designed to make fewer assumptions on children's day to day experiences, which may vary vastly within and across cultures. This is in contrast to many neurobehavioural assessments, which tend to be rooted in object-based infant-adult interaction or play, and thus require careful adaptation for each study setting (Milosavljevic et al., 2019). As shown by a recent review of infant neuroimaging studies (Azhari et al., 2020), the vast majority of neuroimaging research is carried out in high-income countries, with longitudinal study designs still being uncommon. A new generation of studies examining infant development in at-risk populations in low-and middle-income settings may thus provide a set up to investigate neurodevelopmental changes in large, longitudinal cohorts, tapping comprehensive assessment protocols including multiple assessment modalities.

The current study was conducted as part of the Brain Imaging for Global Health (BRIGHT, globalfnirs.org/the-bright-project/) study, which followed two infant cohorts from birth to two years of age living within, or near to, Cambridge in the UK and Keneba, in a rural region of The Gambia. The BRIGHT study protocol encompassed fNIRS and EEG measures, as well as eye tracking and a comprehensive set of neurobehavioural measures (Neonatal Behavioural Assessment Scale, Mullen Scales of Early Learning [MSEL], Language Environment Analysis, Parent Child Interaction). Further, infants’ growth and nutritional status were measured at regular intervals. Using indices from two different neuroimaging paradigms within the BRIGHT study, early analyses by our group have examined developmental changes in habituation and novelty detection in The Gambia using fNIRS (Lloyd-Fox et al., 2019) and EEG (Katus et al., 2020). Our previous work using EEG has relied on an auditory oddball paradigm, in which infants were presented with frequent, infrequent but repetitive and trial unique, novel sounds. This allowed us to compare developmental changes in infants’ response to infrequent but repetitive and trial unique, novel stimuli. Examining neurodevelopmental changes in infants’ ERP between 1 and 5 months of age, we showed that at the group level, infants in the Gambian cohort showed less of a developmental change towards a mature neural novelty response compared to the UK cohort (Katus et al., 2020). Whereas both groups showed large ERP P3 responses to infrequent, repetitive sounds at 1 month of age, only the UK cohort showed a developmental change towards a larger ERP P3 to trial unique sounds at the 5-month age point. The response patterns observed in the Gambian cohort is in contrast to prior reports in the literature from high-income settings, which describe the emergence of a robust novelty-based response (larger ERP P3 to trial unique than infrequent sounds) from around 2 months of age (Otte et al., 2013; van den Heuvel et al., 2015). For example, Otte et al. (2013) report that their 2-month-old participants showed a larger ERP P3 response to trial unique, novel compared to infrequent, repetitive sounds. Similarly, van den Heuvel et al. (2015), report larger ERP P3 responses to novel stimuli in 4-month compared to 2-month-old infants.

We also observed a reduced novelty response in our Gambian cohort in our prior work using fNIRS: infants were presented with repetitions of a sentence of infant directed speech. For 15 repetitions, infants listened to this sentence spoken by a female speaker (habituation trials) before a change to a male speaker occurred (novelty trials). Infants in the Gambian cohort did not show evidence of a neural novelty response at 5 or 8 months of age, in contrast to the UK cohort. Rather, they showed evidence for a continued habituation response spanning all auditory trials, regardless of whether these contained familiar (repetitive) or novel content (Lloyd-Fox et al., 2019). These response patterns are in contrast with earlier work in high-income settings, which documents that even at younger ages (0–3 months) neural response decrements as well as increases in neural activity in response to novel stimuli can be seen (Benavides‐Varela et al., 2011; Bouchon et al., 2015; Nakano et al., 2009).

These findings documenting the development of habituation and novelty detection across infancy using either fNIRS or EEG raise the question of whether both assessment modalities capture the same underlying neurodevelopmental changes, or whether specific underlying mechanisms are tapped by each modality. The implementation of different assessment modalities allows us to simultaneously assess developmental changes in the spatial localisation of responses (e.g., are fNIRS responses differentially localised at the different age points, indicating a shift in neural basis for habituation and novelty detection processes) and function (e.g., is there a change in neural function as measured by EEG across infancy). Using two different paradigms allows us to assess the relationship between lower sensory-level processes (as measured by our EEG paradigm) and higher-level cognitive processes (as measured by subtle speaker change in fNIRS paradigm) across individuals as an indicator of the degree of shared variance captured by the two.

### 1.3 Aims and hypotheses

The aim of the current analysis is to first, assess longitudinal changes in habituation and novelty detection across two neuroimaging paradigms and modalities (EEG and fNIRS) in data collected from the Gambian cohort of the BRIGHT study. Our objective aim is to assess neural metrics across 1-, 5- and 18-months of age in each modality, to examine longitudinal changes in responses in habituation and novelty detection. Our second objective is to assess whether individual differences in habituation and novelty detection measured in EEG are correlated with individual differences in the same domains measured by fNIRS. Lastly, we will assess whether those infants who do show evidence for a habituation response in either modality also show evidence for a robust novelty response. We hypothesise that:

1. Infants will show response decrements in their haemodynamic (assessed by fNIRS) and electrophysiological (assessed by EEG) responses over consecutive trials of a repeated stimulus. Response decrements will occur over a smaller number of trials at the older (5 & 18 months) age points, indicating more efficient habituation processes.
2. Infants will show larger haemodynamic and electrophysiological responses to novel, compared to repeated stimuli. This condition difference will increase with age.
3. Habituation responses measured by EEG and fNIRS will be positively correlated. Equally, novelty responses across the two assessment modalities will show positive correlations.
4. Novelty and habituation will be positively correlated, that is, those infants showing a robust novelty response will also show a robust habituation response across measures.

## 2 Methods

### 2.1 Participants

Participants were recruited into the BRIGHT study antenatally. Expectant women were identified via the Demographic Surveillance System. They were then approached at their antenatal clinic visits to the Keneba field station, situated in the rural West Kiang region of The Gambia, which is part of the Medical Research Council (MRC) Unit The Gambia at the London School of Hygiene and Tropical Medicine (MRCG @ LSHTM, www.mrc.gm). Families indicating an interest in participating provided informed consent during a follow-up home visit. Infants were excluded if born before 37 or after 42 weeks’ gestation, or if they were diagnosed with any neurological deficit during postnatal checks. In total, 204 families were recruited and eligible at the first antenatal visit all of whom were residents of the village of Keneba or surrounding villages in the West Kiang district. Infants were assessed in the home at 7–14 days and then in the clinic at 1-, 5-, 8-, 12-, 18- and 24 months. EEG data were collected at three of these age points, at 1-, 5- and 18- months of age. The BRIGHT protocol also included fNIRS assessments at 8-, 12- and 24 months of infant age, however these will not be described in the current manuscript as no EEG data were collected at these additional age points. For a description of the experimental study setup and adaptation process for fNIRS, EEG and eye tracking see Blasi et al. (2019) and Katus et al. (2019). Only members of the Mandinka ethnic group, who represent the ethnic majority in the West Kiang region of The Gambia (Hennig et al., 2017), were eligible to enrol to avoid confounds arising from translating measures into multiple local languages. Ethical approval was obtained from the joint Gambia Government – MRC Unit The Gambia Ethics Committee (project title ‘Developing brain function for age curves from birth using novel biomarkers of neurocognitive function’, SCC number 1451v2).

### 2.2 EEG study

#### 2.2.1 Stimuli and Design

**Fig. 1:**
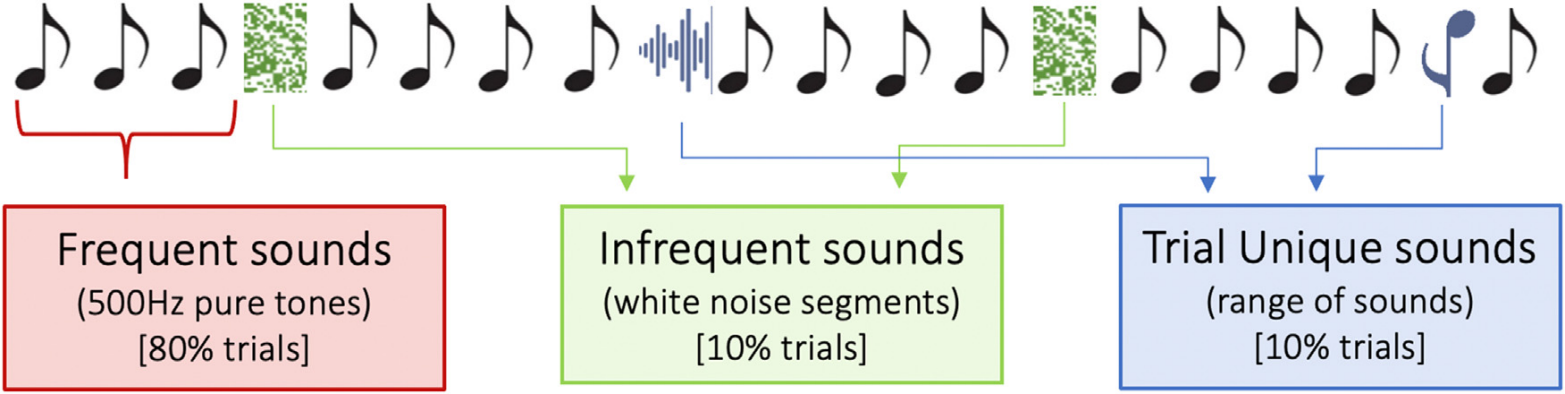
Adapted from Katus et al., 2020. Schematic of stimulus presentation in EEG paradigm. Sounds of three categories were presented: Frequent sounds at a probability of 0.8, consisting of 500 Hz pure tones, Infrequent sounds, presented at 0.1 probability and consisting of short segments of white noise, and Trial Unique sounds, presented at 0.1 probability and consisting of a range of sounds (e.g., vocalisations, digitised syllables, pure tones). Sounds were presented for 100 ms with a 5 ms ramp up and down time, and an ISI of mean length 700 ms, jittered between 650 and 750 ms. (For interpretation of the references to colour in this figure legend, the reader is referred to the web version of this article.)

Procedures for this study are described in Katus et al. (2020). Stimuli for this study were adapted from Kushnerenko et al. (2007). We presented sounds of three different categories: *Frequent* stimuli consisting of 500 Hz pure tones and presented at a probability of 0.8, *Infrequent* sounds, consisting of white noise segments, presented at a probability of 0.1, and *Trial Unique* sounds, consisting of a range of sounds such as clicks, tones, digitised vocalisations and syllables and also presented at a probability of 0.1 (Fig. 1). Sounds were presented for 100 ms with a 5 ms ramp up and down time and an inter-stimulus interval jittered around a mean duration of 700 ms (ranging from 650 to 750 ms). Stimulus presentation was controlled via customised Matlab routines and Psychtoolbox (Brainard, 1997; Kleiner et al., 2007; Pelli, 1997) run from an Apple Macintosh computer. Sounds were played through wireless Sony TMR-RF810R headphones at a fixed volume of 60 dB SPL. In each session, a total of 1000 trials were presented (800 *Frequent*, 100 *Infrequent*, 100 *Trial Unique*).

#### 2.2.2 Apparatus and procedure

The EEG study was performed at the 1-, 5- and 18-month age points. Data were recorded via the Neurolectrics Enobio8 system (https://www.neuroelectrics.com/solutions/enobio/8, sampling rate 500 Hz), with the eight electrodes placed at locations Fz, FC1/2, C1/z/2 and CP1/2 of the 10–20 system. Data were recorded in reference to the infant's left mastoid. At the 1-month age point infants were assessed during sleep, while being held by one of the researchers. At 5 and 18 months, infants were assessed while awake, and sitting on their parent's lap with a researcher quietly interacting with them using toys, bubbles or gesture games. At all age points, sessions were video recorded to allow for identification of movement artefact offline.

#### 2.2.3 Data processing and analysis

Automated Matlab routines were used to pre-process the data: data were bandpass filtered (0.5–30 Hz, blackman, filter order 5500), offset corrected for a 32 ms timing delay and segmented from −200 ms to 800 ms around stimulus presentation. Epochs were rejected via an absolute voltage threshold of >200 µV from minimum to maximum in each epoch. Flatlining epochs (absolute voltage change of <.1 µV) were also discarded. Datasets with <15 valid trials in the *Infrequent* and *Trial Unique* conditions were discarded. Further, to enable habituation analyses described below, datasets with <45 valid trials in the *Frequent* condition were discarded. All results reported were obtained from electrode Fz, which has been shown to be principal for novelty responses (Polich, 2007). At the 1-, 5-, and 18-month age points an average of X̄_1month_ = 62.14 (*SD_1month_* = 14.53), X̄_5month_ = 52.58 (*SD_5month_* = 15.29), X̄_18month_ = 51.29 (*SD_18month_* = 23.15) for the *Infrequent* and *Trial Unique* conditions, and an average of X̄_1month_ = 660.87 (*SD_1month_* = 15.92), X̄_5month_ = 582.95 (*SD_5month_* = 12.19), X̄_18month_ = 541.30 (*SD_18month_* = 28.24) for the *Frequent* condition were retained.

#### 2.2.4 Definition of EEG habituation and novelty detection indices

The present analysis focuses on the mean amplitude of the P3 component over a time window of 250–450 ms (1-month age point) and 200–400 ms (5-and 18-month age points) post stimulus onset. For a detailed description of other ERP components at the 1- and the 5-month age point refer to Katus et al. (2020). Habituation was assessed for responses to the *Frequent* sounds. Averages were extracted for epochs of 15 trials (Familiarisation1_EEG_ - Fam1_EEG_ = trials 1–15, Familiarisation2_EEG_ – Fam2_EEG_ = trials 16–30, Familiarisation3_EEG_ – Fam3_EEG_ = trials 31–45). This epoch length was chosen, as 15 is considered the minimum number of trials for infant EEG data on which it is possible to obtain a robust estimate (DeBoer, Nelson & Scott, 2007), and it therefore allowed us to reliably assess changes in the P3 while not masking habituation effects that may occur within an epoch had more trials been included. We then assessed the percentage change from the first to the third epoch, normalised for individual ERP amplitudes (i.e., Habituation_EEG_ = (Fam1_EEG_-Fam3_EEG_)/Fam1_EEG_). Therefore, higher values indicate higher levels of habituation across trials. Novelty detection was assessed by subtracting the mean amplitude to *Frequent* sounds from the mean amplitude to *Trial Unique* sounds, and normalising this for the amplitude of the *Frequent* sounds (i.e., Novelty_EEG_ = (*Trial Unique* – *Frequent)*/*Frequent*). Prior to this subtraction trial numbers were equalised across the two conditions by selecting a random subset of *Frequent* sounds to match the number of valid trials in the *Trial Unique* condition per infant.

### 2.3 fNIRS study

#### 2.3.1 Stimuli and design

**Fig. 2:**
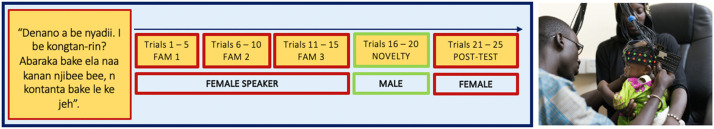
Schematic of stimulus presentation in fNIRS paradigm. Stimuli consisted of 8-second-long sentences of infant directed speech, presented for 25 trials. For the first 15 familiarisation trials (Trials 1–5 = Fam1_NIRS_, Trials 6–10 = Fam2_NIRS_, Trials 11–15 Fam3_NIRS_), the sentence was spoken by a female speaker, followed by 5 trials spoken by a male speaker (Trials 16–20 – Novelty Trials). The final 5 trials were spoken by the same female speaker as for the Familiarisation trials (Post-test Trials). Between each trial, a 10 s silent baseline was presented. Image copyright: Ian Farrell (right hand side photo). (For interpretation of the references to colour in this figure legend, the reader is referred to the web version of this article.)

Procedures for this study are described in Lloyd-Fox et al. (2019). Infants were presented with 8-second-long spoken auditory stimuli of Mandinka infant-directed speech. Two versions of this stimulus were recorded, one spoken by a male and one by a female speaker. Stimuli were recorded at a sampling rate of 48 Khz and edited using Audacity software v2.2.1 to normalise to a peak amplitude of −1 dB SPL and converted from stereo to mono. This study was part of a larger fNIRS protocol, which was presented using customised Matlab routines (Task Engine, sites.google.com/site/taskenginedoc) and Psychtoolbox (Brainard, 1997; Kleiner et al., 2007; Pelli, 1997). Stimuli were presented from an Apple Macintosh computer connected via Logitech Z130 speakers. Sound levels were adjusted to a mean of 60db SPL at the position of the infant's head (ranging from 60.1 to 61.4 dB). Preceding each stimulus was a 10 s silent period which was used as a baseline for the NIRS analyses. A total of 25 trials were presented: 15 trial repetitions of the female speaker, 5 repetitions of the male speaker, and another 5 trials of the female speaker. Trials were then grouped into the following: Trials 1–5 (Familiarisation1_NIRS_ – Fam1_NIRS_), Trials 6–10 (Familiarisation2_NIRS_ – Fam2_NIRS_), Trials 11–15 (Familiarisation3_NIRS_ – Fam3_NIRS_), Trials 16–10 (Novelty Trials), Trials 21–25 (Post-test Trials). The task design is illustrated in Fig. 2.

#### 2.3.2 Apparatus and procedure

**Fig. 3:**
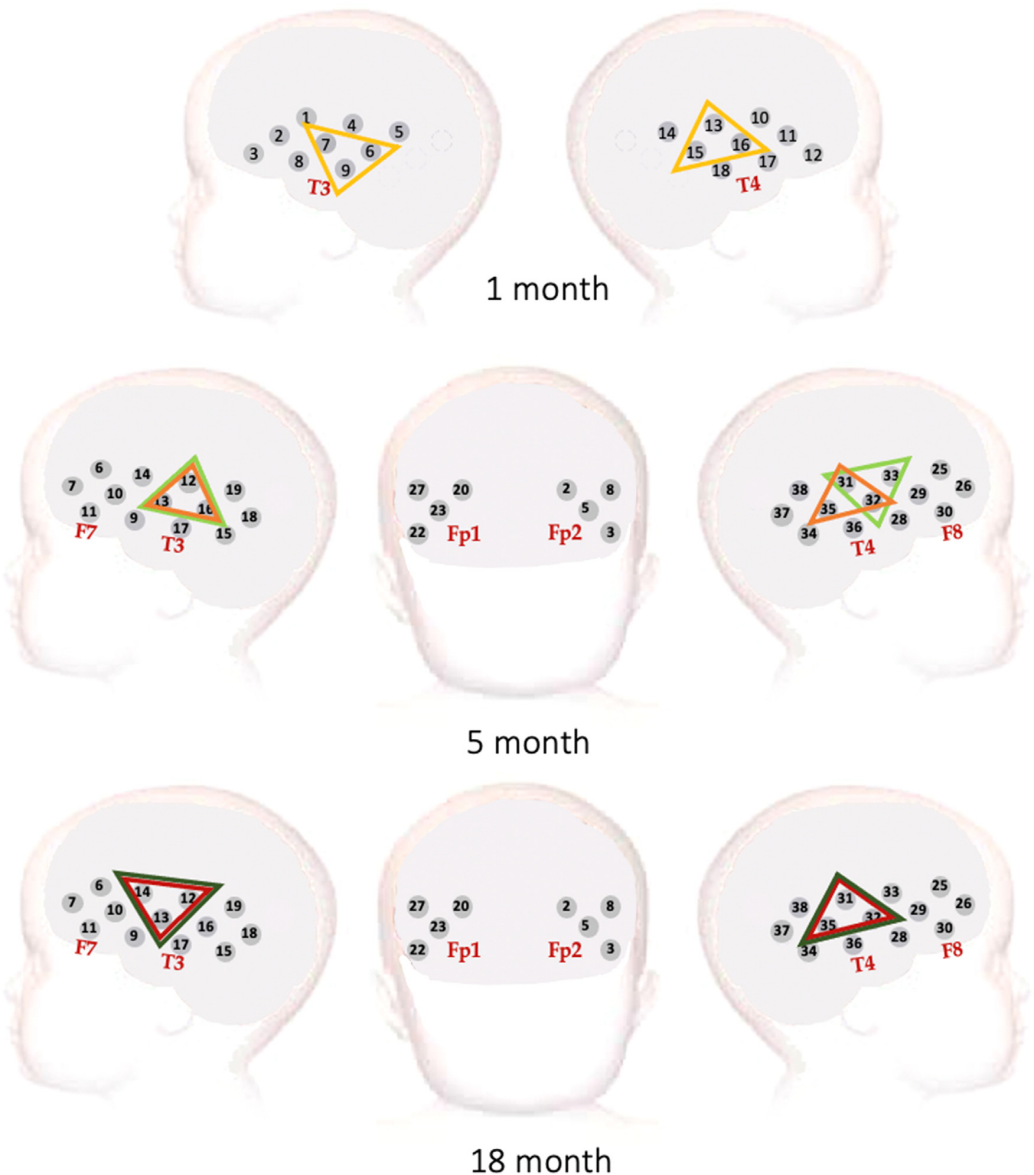
fNIRS channel configuration at the 1-, 5- and 18-month age points. Highlighted are channels contributing to the ROI's at each point as identified by cluster permutation analyses. At the 1-month age point (top panel), a significant ROI based on the Fam1_NIRS_ trials was found over bilateral middle temporal regions (yellow). At the 5-month age point (middle panel), a significant ROI spanning middle to posterior temporal regions was found for the Fam1_NIRS_ trials (orange) and Novelty trials (light green). At the 18-month age point (bottom panel) ROI's were found over middle to posterior temporal regions for the Fam1_NIRS_ (red) and Novelty (dark green) trials. (For interpretation of the references to colour in this figure legend, the reader is referred to the web version of this article.)

The fNIRS habituation and novelty detection study was administered at 1-, 5-, 8-, 12-, 18- and 24-months of infant age. In reference to the age points at which the EEG study was administered, we here present data from the 1-, 5- and 18-month age points. Data were recorded using the Gowerlabs NTS system (Gowerlabs Ltd. London, UK), which emits near infrared light at wavelengths of 780 and 850 nm. Recordings were obtained from 18 channels (9 per hemisphere) at 1 month, and 34 channels (17 per hemisphere) at 5- and 18 months. Source-detector arrays were placed to span the inferior frontal to posterior temporal cortices (Fig. 3). At the 1-month age point, infants were assessed while asleep and being held by one of the researchers. At 5- and 18 months infants were assessed while awake, while sitting on their parent's lap with a researcher holding their attention through quiet presentation of toys or bubbles. Sessions were video recorded to allow for offline identification of excessive movement or social interactions with the parent or the experimenters during the session.

#### 2.3.3 Data processing and analysis

Epochs (Fam1_NIRS_, Fam2 _NIRS_, Fam3 _NIRS_, Novelty Trials, Post-test Trials) with less than three valid trials per infant were disregarded from group-level analyses. Datasets with one or more non-valid familiarisation epochs were excluded from further analysis.

Light attenuation measures for each source-detector pair were converted into changes in oxy-haemoglobin (HbO_2_) and deoxy-haemoglobin (HHb) in µM to obtain a measure of neural activity (Kocsis et al., 2006). Data were pre-processed using customised Matlab routines in an analysis pipeline similar to other infant studies (Gervain et al., 2011; Lloyd-Fox et al., 2010). First, channels with readings of less than 3^e-4^ were excluded. This value was chosen based on previous experience with the NTS system, and ensures the exclusion of channels for which insufficient NIR light is reaching a detector (e.g., due to the detector being blocked, or unclipped from the array). Secondly, channels exceeding the maximum acceptable difference of 0.2 between the coefficients of variation in the attenuation readings for the 780 and 850 nm wavelengths per channel were discarded, to prevent inclusion of channels in which noise differently affected the two wavelength readings. Lastly, power spectrum density analyses of the raw signal were used to discard channels which showed strong activation in frequencies unrelated to neural activity. Raw intensity data were then inspected according to the above criteria for each infant, using automated quality control scripts. Infants with fewer than 60% of valid channels were excluded. Data were divided into blocks consisting of 4 s preceding the auditory stimulus (baseline), the auditory stimulus itself and the following baseline trial. For each block, attenuation data were then detrended using a linear fit between the first and the last 4 s of the block.

Following preprocessing, attenuation data were converted into changes in concentration of HbO_2_ and HHb (µM) using the modified Beer Lambert law (Delpy et al., 1988). The conversion assumed an age-dependant differential pathlength factor (DPF) calculated from Duncan et al. (1995). After the conversion, a second round of artefact rejection was conducted on a trial-by-trial basis (per channel), to identify motion artefact (concentration changes of a predefined threshold of +/- 3.5 µM during the baseline or +/- 5 µM during the experimental trial were excluded).

Offline coding of infant behaviours such as active interaction with the parent or the experimenter, fussiness or distress were coded as invalid sections of the session. For each trial, if such behaviours exceeded 40%, the trial was marked as invalid. This is in line with previous infant studies using a different protocol involving visual and auditory stimulation, where the rejection threshold was set to 40% of the stimulation period (for an example see Lloyd-Fox et al., 2014).

Trials and channels surviving the rejection were retained for further analyses. Overall the numbers of trials retained across the five epochs per age point were Fam1_NIRS_: X̄_1month_ = 4.89 (*SD_1month_ =* 0.41), X̄_5month_ = 4.96 (*SD_5month_ =* 0.22) X̄_18month_ = 4.73 (*SD_18month_ =* 0.58), Fam2_NIRS_: X̄_1month_ = 4.90 (*SD_1month_ =* 0.37), X̄_5month_ = 4.99 (*SD_5month_ =* 0.09) X̄_18month_ = 4.81 (*SD_18month_ =* 0.46), Fam3_NIRS_: X̄_1month_ = 4.88 (*SD_1month_ =* 0.44), X̄_5month_ = 4.99 (*SD_5month_ =* 0.12) X̄_18month_ = 4.68 (*SD_18month_ =* 0.70), Novelty Trials: X̄_1month_ = 4.82 (*SD_1month_ =* 0.61), X̄_5month_ = 4.93 (*SD_5month_ =* 0.41) X̄_18month_ = 4.49 (*SD_18month_ =* 1.04), Post test: X̄_1month_ = 4.72 (*SD_1month_ =* 0.78), X̄_5month_ = 4.93 (*SD_5month_ =* 0.42) X̄_18month_ = 4.42 (*SD_18month_ =* 1.01). Trials were then averaged across each epoch and infants, yielding a time course of the mean concentration change in HbO_2_ and HHb per channel. While based on pair-wise comparison the trial numbers differed between age points (due to generally higher noise levels in older infants), these differences were not sufficient to lead to a violation of the model assumptions: for example, our RM-ANOVA's sphericity, which could be affected by differences in trial number via differences in magnitude of the standard deviation, was not violated. To not further reduce the amount of available data, we therefore did not even out differences in trials numbers across age points.

#### 2.3.4 Definition of fNIRS habituation and novelty detection indices

For each averaged epoch, a temporal window of 8–12 s from stimulus onset was selected, in order to include the range of maximum concentration changes observed across all infants for the HbO_2_ and HHb responses. This window is consistent with the previously published analysis on a subset of the NIRS data presented here (Lloyd-Fox et al., 2019). The averaged time course of the signals within this window were then compared to responses across the average of the final four seconds preceding the auditory stimulus (baseline). Either a significant increase in HbO_2_ or a significant decrease in HHb (but not a simultaneous significant increase or decrease of both signals) was accepted as an indicator of neural activity, in line with prior research (Lloyd-Fox et al., 2010). Two-tailed t-tests of the HbO_2_ and HHb change averaged across the time window of interest were used to identify active channels. False-discovery rate (FDR, Benjamini and Hochberg, 1995) correction was implemented to resolve multiple comparisons issues.

For a more data-driven approach, resulting t-values were then entered into a cluster-based permutation analysis (Maris and Oostenveld, 2007). This nonparametric approach was used to select the region of interest (ROI) by adopting anatomically informed conditions on the clusters being considered (i.e. three non-aligned channels per cluster). Selection of this method provided a path to finding ROIs from a paradigm and age ranges not previously documented in the literature. Furthermore, it also helped confirm results from the t-tests, as this method offers a solution to the multiple comparisons issue, which appears when data is collected simultaneously from multiple points (Maris and Oostenveld, 2007). The cluster-based permutation analysis had been used on a subset of the NIRS data presented here and included in a previous publication (Lloyd-Fox et al., 2019) and has also been applied to infant data in other works (Abboub et al., 2016; Benavides-Varela and Gervain, 2017; Ferry et al., 2016). First, channels on each array were arranged in triangulated clusters, each containing three nearest-neighbouring channels. This resulted in 58 pre-defined clusters in total. Each cluster was assigned a t-value, calculated by adding the individual t-values of its channel components, as computed in the step described above for the Fam1_NIRS_ condition (relative to baseline) within a window of 8–12 s post stimulus onset. Then, the mean signal change was randomized by participant and channel, and new t-values were calculated per channel and summed within each cluster to obtain the new cluster t-value. This randomisation and calculation of cluster t-values was repeated 1000 times to generate a cluster probability curve of t-values. In total, *N* = 1000 permutations was chosen based on previous fNIRS research groups using this method (Abboub et al., 2016; Benavides-Varela and Gervain, 2017). The t-value of each cluster candidate was then tested to see whether it was significantly different from chance by calculating its p-value as the area under its probability distribution to the right of the cluster t-value. The process was repeated for all candidate clusters. At each time point, the cluster within each array (left and right) with the most significant p-value was selected.

Given that the clusters identified in each hemisphere were over similar regions, and there were no a-priori hypotheses about differential hemispheric habituation and novelty effects (as responses were found in both hemispheres in previous research; Benavides-Varela et al., 2011, Nakano et al., 2009), these were then combined across hemispheres to generate a primary bilateral ROI for the main analyses. Cluster-based permutation analyses were repeated for the Novelty condition, to investigate whether the location of the Novelty response (compared to baseline) was in a similar region to the response to Fam1. At the 1-month age point, no channels showed any significant activation to the Novelty response; at the 5-month age point Fam1_NIRS_ and Novelty ROIs differed by one channel only; and at 18 months, ROIs or both conditions were identical.

Signals from each of the channels included in the ROIs were inspected for meaningful neuronal response was based on both HbO_2_ and HHb (i.e., significant increase in HbO2, significant decrease in HHb or both). Once we identified which ROI showed meaningful neuronal activation based on both chromophores, we focussed our statistical analyses of habituation, novelty detection and comparison with the EEG signal on HbO_2_ responses. This was done as HbO2 has been found to be the more robust measure in our past work (Blasi et al., 2014). To examine habituation, we obtained the differences HbO2 responses between Fam1_NIRS_ and Fam3_NIRS_, normalised by Fam1_NIRS_ (i.e., Habituation_NIRS_ = (Fam1_NIRS_ – Fam3_NIRS_)/Fam1_NIRS_). Novelty detection was assessed via subtracting Fam3_NIRS_ from Novelty trials and dividing this by Fam3_NIRS_ (i.e., Novelty_NIRS = (_Novelty – Fam3_NIRS_)/Fam3_NIRS_).

### 2.4 Statistical analyses

First, we examined time-course responses for our fNIRS and EEG measures. We then modelled mean amplitudes for the ERP P3 component by condition (*Frequent / Infrequent / Trial Unique*) and age (1 month / 5 months / 18 months) longitudinally in a repeated measures ANOVA. For the fNIRS responses, we modelled the mean haemodynamic change during the 8–12 s time window post stimulus onset by epoch (Fam 1 / Fam3 / Novelty / Post test) and age (1 month / 5 month / 18 months) in a repeated measures ANOVA. Significant main effects were followed up by paired t-tests, resulting p-values were FDR corrected. We hereby included all three age points in a joint analysis, even though infants at 1 month were assessed asleep, in contrast to both other age points. This decision was taken to be able to model longitudinal trends in these neural responses, and in part justified by previous analyses into the effect of state changes by our group. In a previous analysis (Katus et al., 2020), we found that neural responses did not differ significantly between infants tested asleep vs. awake at 5 months of age. We further found that the developmental change between 1 and 5 months did not differ for those who changed state between age points and those who were assessed asleep both times. While this is not to negate the impact of state, we found that for the specific metrics observed state did not seem to have a statistically significant effect. As no such analyses could be conducted for the NIRS data, we opted to model the effect of condition in a repeated measures ANOVA per age point, to not conflate possible developmental effects with the effect in state change between 1 month and the other age points.

Second, we examined developmental changes in infants’ habituation and novelty detection responses per imaging modality. To assess habituation, we separately modelled our habituation indices (Habituation_EEG_ and Habituation_NIRS_) and novelty indices (Novelty_EEG_ and Novelty_NIRS_) in a repeated measures ANOVA by age (1 month / 5 month / 18 month).

Third, we examined one-tailed Pearson correlations to investigate associations between the EEG and NIRS metrics of habituation and novelty detection per age point. Last, we assessed whether infants who show strong habituation responses also show strong novelty responses, by stratifying habituation correlations by novelty responses and vice versa. To this end, infants’ habituation and novelty responses were dichotomised (Habituation < 0 coded as 0, Habituation > 0 coded as 1, and likewise for Novelty responses): infants could score 0 (no habituation/novelty detection in either NIRS or EEG), 1 (habituation/novelty detection in either NIRS or EEG or 2 (habituation/novelty detection in both NIRS and EEG). We then examined what proportion of infants scoring high on novelty detection also showed high scores in habituation and vice versa.

## 3 Results

**Fig. 4:**
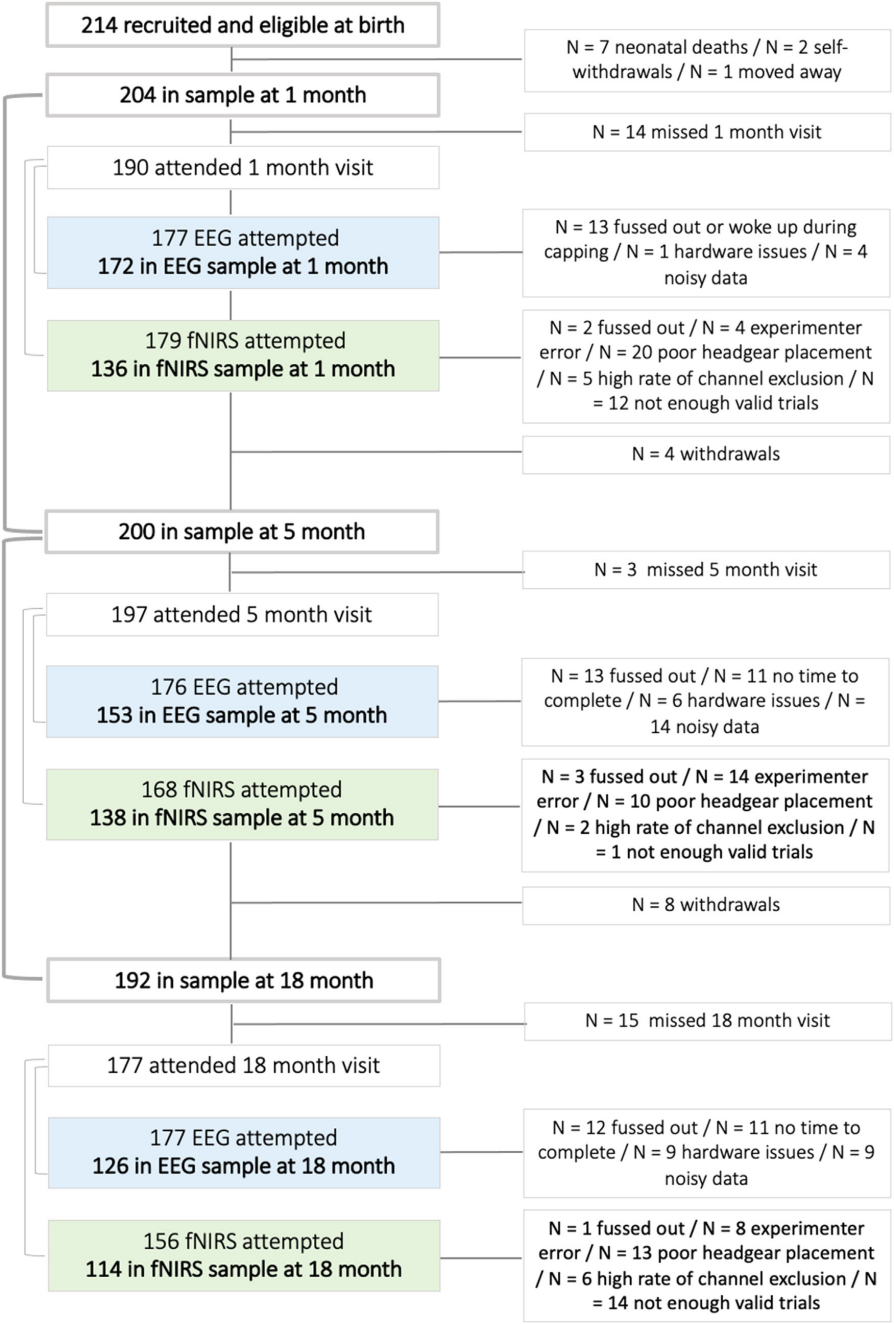
Rates of data exclusion / retention at the 1-, 5- and 18-month age point and reasons for exclusion. (For interpretation of the references to colour in this figure legend, the reader is referred to the web version of this article.)

Prior to the main analyses examining correlations across our fNIRS and EEG paradigm (Section 3.3), we conducted checks on data retention and quality, as well as examinations of within-modality developmental changes. For a proportion of infants, data were missing for one of the following reasons (Fig. 4): 1) infants passing away, discontinuing the study or missing a study visit, 2) infants not tolerating placement of the fNIRS or EEG cap or being too fussy to record sufficient data, 3) improper headgear placement, 4) data were found to be too noisy, for example due to motion artefact, 5) technical or experimenter error.

Descriptive statistics can be found in Table 1. No differences were observed between those infants included vs. excluded in the present analyses with regard to their sex, age, weight, head circumference and length (*p* > 0.172).

**Table 1:** Descriptive statistics of infant age, sex, and anthropometric measures for infants included and excluded in further analyses.

	**EEG**						**fNIRS**					
	1-month		5-months		18-months		1-month		5-months		18-months	
	Included	Excluded	Included	Excluded	Included	Excluded	Included	Excluded	Included	Excluded	Included	Excluded

**Characteristics**												

Sex (m/f)	87/85	17/15	76/77	22/21	67/59	26/37	76/60	27/40	72/66	29/32	54/60	39/36
	x̄ ± SD	x̄ ± SD	x̄ ± SD	x̄ ± SD	x̄ ± SD	x̄ ± SD	x̄ ± SD	x̄ ± SD	x̄ ± SD	x̄ ± SD	x̄ ± SD	x̄ ± SD
Age (days)	44.31 ± 28.01	42.72 ± 26.40	161.108 ± 10.52	158.79 ± 10.43	573.61 ± 39.01	564.39 ± 38.91	42.01 ± 29.14	44.91 ± 28.96	158.91 ± 9.89	162.86 ± 11.42	561.97 ± 39.56	571.72 ± 39.65
Weight (kg)	4.391 ± 0.624	4.274 ± 0.573	6.821 ± 0.814	6.791 ± 0.717	9.52 ± 0.921	9.49 ± 1.031	4.221 ± 0.471	4.467 ± 0.615	6.792 ± 0.783	6.924 ± 0.835	9.63 ± 1.052	9.39 ± 0.891
Length (cm)	54.132 ± 1.912	52.912 ± 2.811	63.972 ± 1.967	64.092 ± 2.245	79.413 ± 3.12	78.201 ± 2.92	51.917 ± 2.275	55.395 ± 1.984	65.392 ± 2.192	60.214 ± 2.423	79.912 ± 3.01	78.261 ± 3.29
Head circumference (cm)	36.411 ± 2.113	37.121 ± 1.981	42.351 ± 1.425	41.663 ± 1.342	46.831 ± 1.653	47.318 ± 1.583	37.204 ± 1.211	36.123 ± 1.922	42.938 ± 1.623	40.916 ± 1.562	48.391 ± 1.284	45.326 ± 1.572

**Anthropometric z-scores**												

WAZ	−0.498 ± 0.871	−0.531 ± 0.918	−0.653 ± 0.892	−0.625 ± 0.916	−0.971 ± 0.921	−1.051 ± 0.957	−0.521 ± 0.973	−0.491 ± 0.981	−0.692 ± 0.928	−0.651 ± 0.914	−1.0821 ± 1.027	−1.0271 ± 1.205
LAZ	−0.913 ± 0.915	−0.832 ± 0.899	−0.5986 ± 0.983	−0.641 ± 0.893	−1.112 ± 0.792	−1.372 ± 1.392	−0.877 ± 0.941	−0.824 ± 0.951	−0.611 ± 0.951	−0.613 ± 0.941	−1.148 ± 0.810	−1.319 ± 1.124
HCZ	−0.614 ± 0.798	−0.579 ± 0.893	−0.712 ± 0.974	−0.761 ± 0.951	−0.942 ± 0.9752	−0.892 ± 0.891	−0.528 ± 0.913	−0.519 ± 0.893	−0.749 ± 0.897	−0.751 ± 0.956	- 1.021 ± 1.129	−0.823 ± 0.975
WLZ	0.427 ± 0.980	0.341 ± 1.126	−0.261 ± 0.981	−0.245 ± 1.021	−0.812 ± 1.072	−0.741 ± 0.985	0.386 ± 1.314	0.321 ± 0.986	−0.26 ± 1.21	−0.269 ± 0.983	−0892 ± 1.042	−0.856 ± 1.032

Note. No differences between infants included and excluded in analyses were seen regarding sex, age, weight, length, head circumference, WAZ = weight-for-age z-scores, LAZ = length-for-age z-scores, HCZ = head circumference-for-age z scores or WLZ = weight-for-length z scores. (all *p* > 0.172).

### 3.1 Developmental change in EEG and fNIRS response 1–18 months

#### 3.1.1 Longitudinal ERP results 1–18 months

**Fig. 5:**
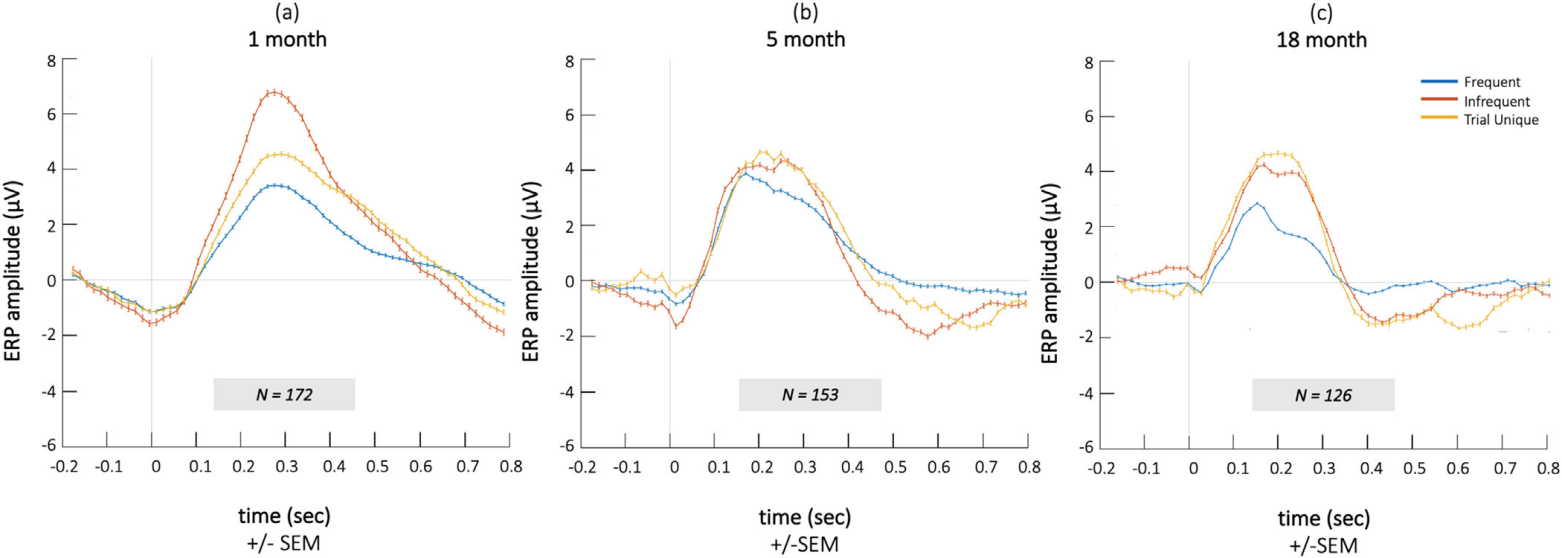
ERP responses at 1-month (a), 5-months (b) and 18-months (c) of age for *Frequent* (blue), *Infrequent* (red) and *Trial Unique* (yellow) sounds. Here, time courses of all infants contributing valid data for each cross-sectional age point are included. Figures including only infants contributing EEG data to all three age points (*N* = 74) can be found in Supplementary Figure 1. (For interpretation of the references to colour in this figure legend, the reader is referred to the web version of this article.)

The ERPs for all infants contributing valid data at the 1-, 5- and 18-month age point are displayed in Fig. 5.

The repeated measures ANOVA showed significant main effects for condition (*F_2,__146_* = 14.266, *p* < 0.001, *n_p_^2^* = 0.163), but not age (*F_2,__146_* = 2.436, *p* = 0.091, *n_p_^2^* = 0.032). We also found an age* condition interaction effect (*F_4,__292_* = 3.753, *p* = 0.006, *n_p_^2^* = 0.049), which was followed up by post-hoc comparisons: 1-month-old infants showed a large ERP P3 component in response to *Infrequent*, white noise sounds compared to *Frequent* (*t_171_ =* 8.204, *p_FDR_* < 0.001, *d* = 0.626) and *Trial Unique* (*t_171_ =* 3.929, *p_FDR_* < 0.001, *d* = 0.3) stimuli, indicating the absence of a novelty-based response at group level. At 5 months, infants showed larger P3 responses to *Infrequent* compared to *Frequent* (*t_152_* = 3.556, *p_FDR_* = 0.001, *d* = 0.287) and Trial Unique compared to Frequent sounds (*t_152_ =* 3.722, *p_FDR_* < 0.001, *d* = 0.301), but responses did not differ between Infrequent and *Trial Unique* sounds, indicating that at group level infants did not show a consistent novelty response. At 18 months, infants showed a novelty response on group level, indicated by higher P3 amplitudes to *Trial Unique* compared to *Frequent* (*t_125_ =* 2.436, *p_FDR_* = 0.016, *d* = 0.217) and *Infrequent* sounds (*t_125_ =* 2.385, *p_FDR_* = 0.019, *d* = 0.212).

#### 3.1.2 fNIRS results 1–18 months

**Fig. 6:**
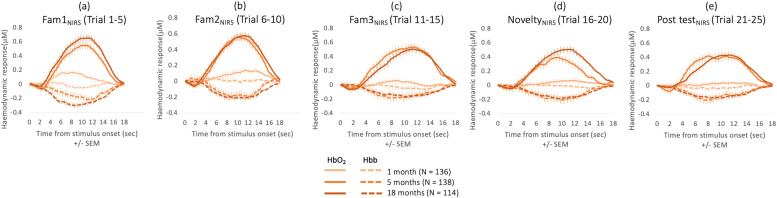
fNIRS time courses at 1 month (light orange), 5 months (orange) and 18 months (dark orange) across Fam1_NIRS_ (a), Fam2_NIRS_ (b), Fam3_NIRS_ (c), Novelty (d) and Post-test (e) epochs. Here, time courses of all infants contributing valid data for each cross-sectional age point are shown. Figures including only infants contributing fNIRS data to all three age points (*N* = 60) can be found in Supplementary Figure 2. (For interpretation of the references to colour in this figure legend, the reader is referred to the web version of this article.)

ROI's for each age point and hemisphere are displayed in Fig. 3. For all three age points, responses were localised at bilateral middle temporal structures. fNIRS time courses per age point (including all infants contributing valid data for each individual age point) are represented in Fig. 6.

For this paradigm, we anticipated a response pattern of: (i) large amplitude change in the fNIRS signals at Fam1_NIRS_; (ii) diminishing amplitude change at Fam2 _NIRS_ and Fam3 _NIRS_ (trials 6 to 10 and 11 to 15); (iii) increased amplitude response at Novelty (trials 16 to 20) compared to Fam3 _NIRS_; and (iv) diminished response at Post test (trials 21 to 25) compared to Novelty _NIRS_ trials (Lloyd-Fox et al., 2019; Nakano et al., 2009). Sleeping 1-month-olds presented smaller amplitude HbO_2_ change in the posterior temporal ROI during Fam1_NIRS_, (trials 1 to 5) compared to the 5-month and 18-month age points. At the 1-month age point, significant increases in oxyhaemoglobin to Fam1_NIRS_ trials were detected on channels spanning both hemispheres; however, none of the channels showed significant activation to the Novelty trials at this time point. A repeated measures ANOVA analysis did not reveal an epoch effect.

At the 5-month age point, we found a significant epoch effect (*F_4,__500_* = 2.887, *p* = 0.022 and *n_p_^2^* = 0.023), driven by a significantly larger response to Fam1_NIRS_ compared to Novelty_NIRS_ trials (*t_132_ =* 2.533, *p_FDR_* = 0.012, *d* = 0.27); and a significantly larger response to Fam2_NIRS_ compared to Novelty trials (*t_132_* = 1.923, *p_FDR_ =* 0.035, *d* = 0.184). This indicates that instead of a novelty response to the change in speaker, infants at this age showed a continued habituation response spanning all trials regardless of stimulus condition.

At 18 months, there was a strong epoch effect (*F_4,__372_* = 5.974, *p <* 0.001 and *n_p_^2^* = 0.060), driven by a significantly larger response for Fam1_NIRS_ compared to Fam2_NIRS_ trials (*t_113_* = 3.765, *p_FDR_* < 0.001, *d* = 0.353) and Fam3_NIRS_ (*t_112_ =* 4.727, *p_FDR_* < 0.001, *d* = 0.445), indicating the emergence of a habituation response. We also found a significant Fam1_NIRS_ > Novelty effect (*t_103_* = 3.552, *p_FDR_* = 0.001, *d* = 0.37) indicating the emergence of a novelty response; and a significant Fam1_NIRS_ > Post test effect (*t_97_ =* 3.678, *p_FDR_* < 0.001, *d* = 0.41).

As the response to Novelty at 18 months of age appears stronger and with a different time profile than at 5 months, post-hoc analyses were performed. Paired *t*-test (FDR corrected) with the subset of 72 infants with valid data at 5 and 18 months reveal no significant difference between the Novelty response at 5 and 18 months within the 8 to 12 s post stimulus onset time window (*t_71_* = 0.766, *p_FDR_ =* 0.446). However, at a slightly later time window from 10 to 14 s post stimulus onset, the Novelty response at 18 months remained significantly larger (*t_71_* = 2.019, *p_FDR_ =* 0.047, *d* = 0.31) indicating a more protracted and sustained response overall.

### 3.2 Longitudinal habituation and novelty responses from 1 to 18 months

**Fig. 7:**
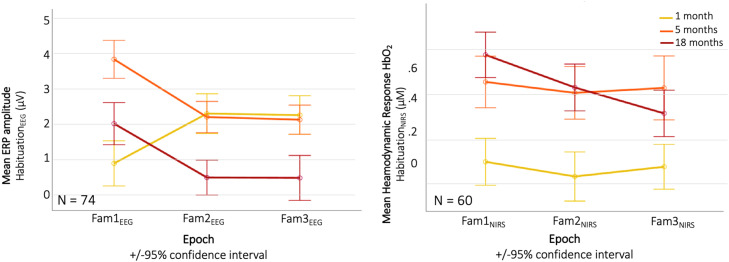
Longitudinal EEG and fNIRS responses across repeated trials per age point. Here, only infants contributing data at all age points are included. (For interpretation of the references to colour in this figure legend, the reader is referred to the web version of this article.)

Habituation profiles for the NIRS and EEG paradigm are displayed in Fig. 7.

**Fig. 8:**
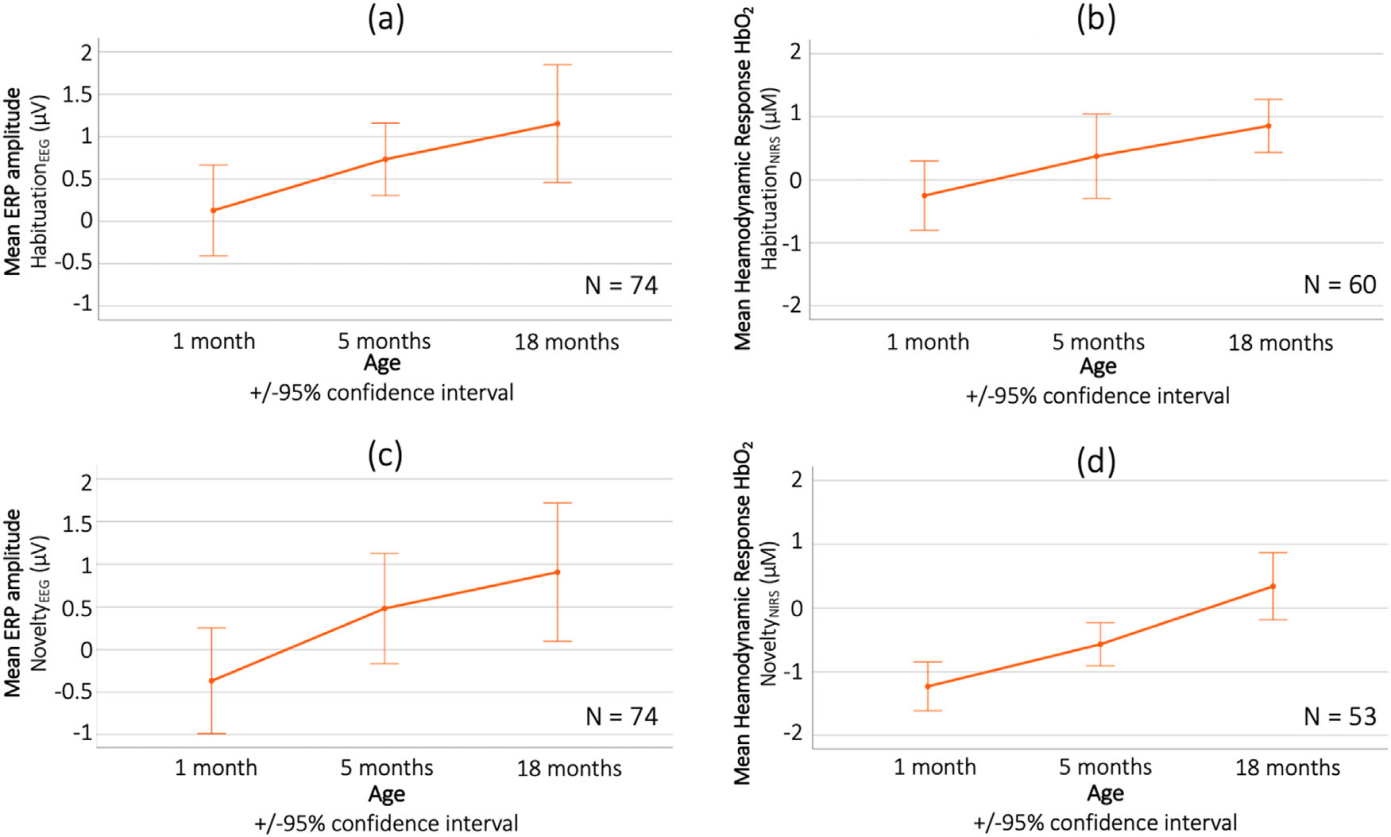
Longitudinal Habituation (top row) and Novelty (bottom row) responses during the EEG (left) and fNIRS (right) paradigm across the 1-, 5- and 18-month age points. Here, only infants contributing data at all age points are included. (For interpretation of the references to colour in this figure legend, the reader is referred to the web version of this article.)

To statistically assess developmental changes in the EEG habituation response, we modelled the Habituation_EEG_ index in a repeated measured ANOVA by age (1 month / 5 month / 18 months), showing a main effect (*F_2,__146_* = 3.167*, p = 0*.045, *η_p_^2^* *=* *0*.042). Post hoc tests showed that this was driven by an increase in stronger habituation responses as 5 months compared to 1 month (*t_112_* = 2.408, *p_FDR_* = 0.018, *d* = 0.217). We also modelled the Habituation_NIRS_ index by age, showing a main effect (*F_2,__118_* = 3.878*, p = 0*.023, *η_p_^2^* *=* *0*.062), driven by an increase in habituation response between 1 month and 5 months (*t_88_* = 3.106, *p_FDR_* = 0.003, *d* = 0.329) and between 1 month and 18 months (*t_82_* = 4.809, *p_FDR_* < 0.001, *d* = 0.528). Results from the EEG and fNIRS habituation analysis are displayed in Fig. 8 (top row).

As for the developmental change in novelty detection, we modelled the Novelty_EEG_ and Novelty_NIRS_ indices in two separate repeated measures ANOVAs with within factor age (1 month / 5 month / 18 month). For the EEG, we found a main effect for age (*F_2,__146_* = 3.359*, p = 0*.037, *η_p_^2^* *=* *0*.044), driven by larger novelty responses at 5 months compared to 1 month (*t_112_* = 3.103, *p_FDR_* = 0.002, *d* = 0.28) and at 18 months compared to 1 month (*t_94_* = 2.472, *p_FDR_* = 0.015, *d* = 0.254). For the fNIRS, we found a main effect (*F_2,__104_* = 14.5*, p < 0*.001, *η_p_^2^* *=* *0*.218), driven by a trend towards larger novelty responses at 5 months compared to 1 month (*t_83_* = 1.954, *p_FDR_* = 0.054, *d* = 0.213) and significantly larger responses at 18 months compared to 5 months (*t_70_* = 2.204, *p_FDR_* = 0.031, *d* = 0.262). Results from the EEG and fNIRS novelty analysis are displayed in Fig. 8.

### 3.3 Cross-sectional correlations of EEG and fNIRS responses at 1, 5 and 18 months

**Fig. 9:**
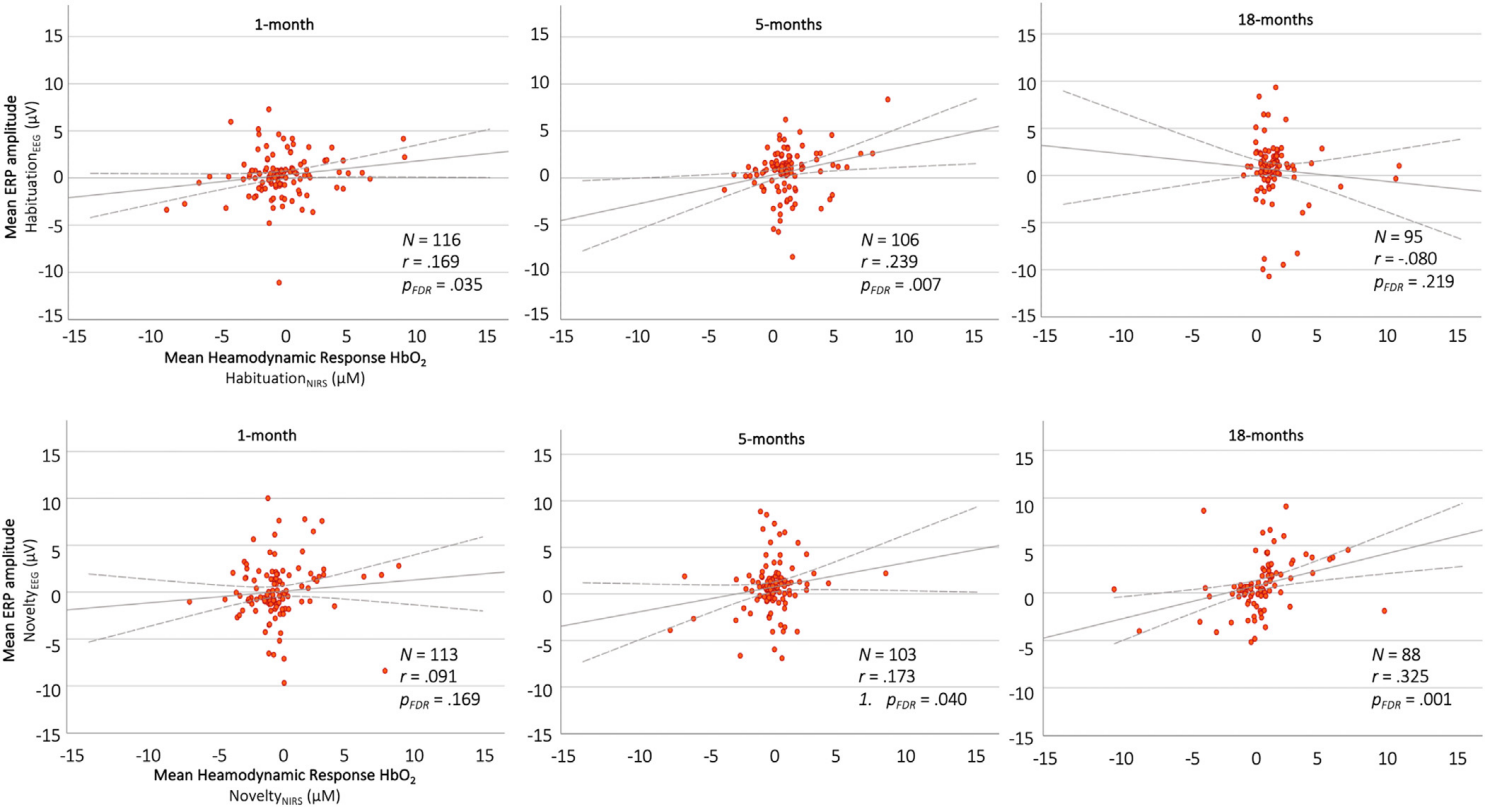
Correlations between EEG (y-axis) and fNIRS (x-axis) habituation (top row) and novelty (bottom row) metric for the 1-, 5-, and 18-month age points. Each data point represents an individual participant's neural response on the EEG and fNIRS paradigm. (For interpretation of the references to colour in this figure legend, the reader is referred to the web version of this article.)

To assess the hypothesized positive correlations between habituation and novelty responses on the EEG and fNIRS paradigm, one-tailed Pearson correlations between the corresponding indices were run per age point, results of which were corrected for multiple comparisons via FDR corrections. For the habituation indices, significant positive correlations were observed at the 1 month and the 5 month age points (1 month: *N* = 116, *r* = 0.169, *p_FDR_* = 0.035, *R^2^* = 0.029; 5 months: *N* = 106, *r* = 0.239, *p_FDR_* = 0.007, *R^2^* = 0.057), but not at the 18 months age point (18 months: *N* = 95, *r* = −0.080, *p_FDR_* = 0.219, *R^2^* = 0.001). For the novelty indices, a positive correlation was found for the 5 month and the 18 month age points (5 months: *N* = 103, *r* = 0.173, *p_FDR_* = 0.040, *R^2^ =* 0.029; 18 months: *N* = 88, *r* = 0.325, *p_FDR_* =0.001, *R^2^ =* 0.106), but not for the 1-month age point (1 month: *N* = 113, *r* = 0.091, *p_FDR_* = 0.169, *R^2^ =* 0.008). All correlations are visualised in Fig. 9.

### 3.4 Cross sectional associations between habituation and novelty responses at 1, 5 and 18 months

We lastly explored whether participants’ habituation responses were associated with their novelty detection responses in either imaging modality. Infant's responses were dichotomised for their habituation and novelty responses, where responses < 0 was allocated a score of 0, and responses > 0 was allocated a score of 1. A sum scores was obtained, where infants could score either: 0 – indicating the absence of novelty responses in both EEG and fNIRS; 1 – indicating a novelty response in either modality; or 2 – indicating a novelty response in both modalities. Correlation analyses were then stratified by the novelty detection sum score (for the habituation analysis) and the habituation sum score (for the novelty analysis).

**Fig. 10:**
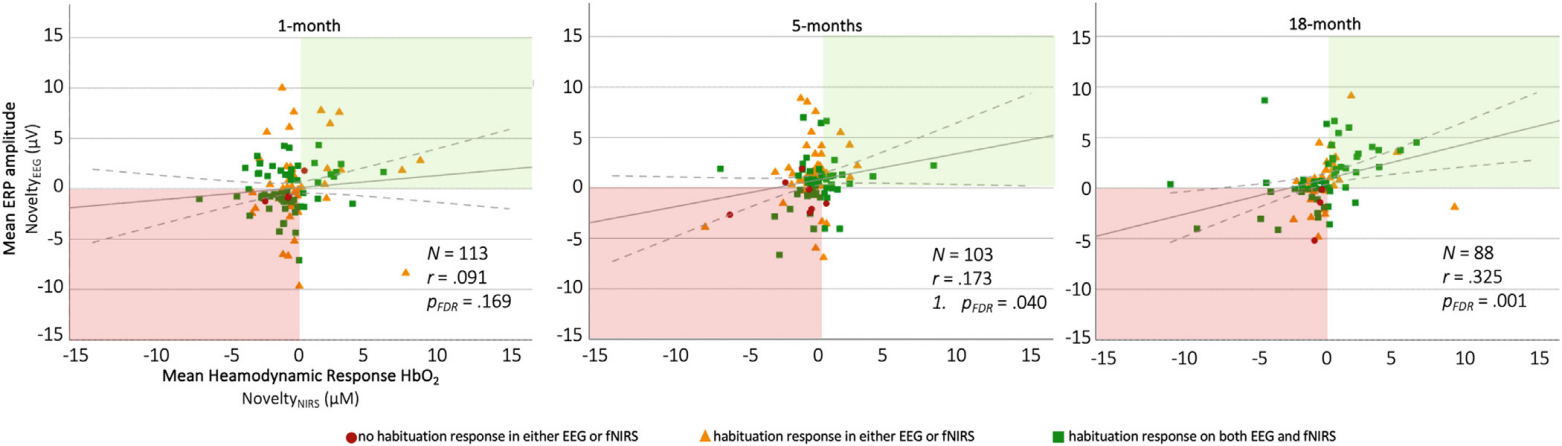
Correlations between EEG (y-axis) and fNIRS (x-axis) novelty metric stratified by habituation responses for the 1-, 5-, and 18-month age points. Each data point represents an individual participant's neural response on the EEG and fNIRS paradigm. A larger number of infants who show a habituation response in either NIRS or EEG (yellow triangles) or both NIRS and EEG (green square) also show a novelty response in both NIRS and EEG (top right quadrant) at the 5 and the 18 month age points. (For interpretation of the references to colour in this figure legend, the reader is referred to the web version of this article.)

**Fig. 11:**
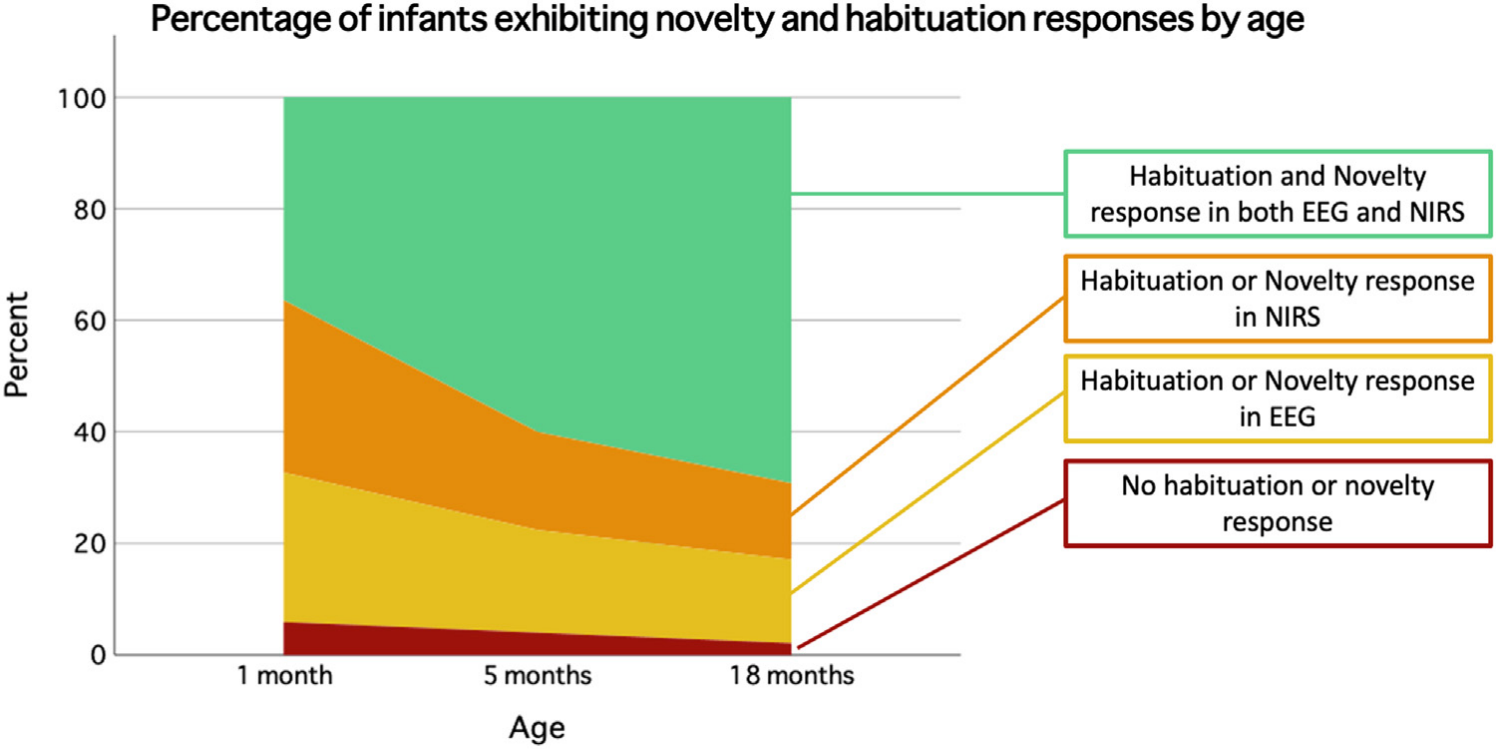
Breakdown of infants showing novelty or habituation responses per modality (NIRS/EEG) and age point (1, 5, 18 months). As can be seen, the proportion of infants showing robust novelty and habituation responses in both NIRS and EEG increases with age. The proportion of infants who show a habituation or novelty response in only NIRS or EEG decreases with age, as does the proportion who shows no novelty or habituation response in either modality. (For interpretation of the references to colour in this figure legend, the reader is referred to the web version of this article.)

As can be seen in Fig. 10, a larger proportion of infants who scored 1 or 2 on their habituation sum score also obtained higher novelty values for both NIRS and EEG. Across the three age points, the proportion of infants who showed a habituation and novelty response in both EEG and fNIRS increased, whereas the proportion not showing any novelty or habituation responses decreased. A full breakdown of the percentages of infants’ novelty responses relative to their habituation responses per EEG and fNIRS can be found in Fig. 11.

## 4 Discussion

The current study is the first to present correlations in two habituation and novelty detection paradigms measured across two neuroimaging modalities (EEG auditory oddball and fNIRS infant-directed speech processing paradigm) across a longitudinal sample spanning the transition from the neonatal period to toddlerhood. As such, the study provides a first demonstration of the benefits of longitudinal, cross-modal protocols to define robust metrics of early neural specialisation. The study adds to our previous work by 1) describing positive correlations between habituation and novelty detection at three longitudinal age points from 1 to 18 months of life, thus covering a crucial window of neurodevelopment, and 2) assessing correlations of neurodevelopmental indices across two increasingly used assessment modalities. Common developmental trends across both the fNIRS and the EEG paradigm suggest that our results are not a specific correlate of a single method or paradigm, but that both methods are measuring the same underlying neuronal response.

### 4.1 Longitudinal habituation and novelty responses in fNIRS and EEG

Across both the EEG and the fNIRS paradigm we found habituation responses increased with age. Specifically, for the EEG paradigm, neural response decrements were significantly higher at 5 months compared to 1 month of age. For the fNIRS paradigm, response decrements were higher at 5 and 18 months, compared to the 1-month age point. The fNIRS responses were consistently localised to fNIRS channels covering infants’ middle temporal areas, with no developmental change in localisation seen across the observed age range. In terms of the novelty detection responses, we observed strikingly similar developmental gains in both modalities: EEG responses increased significantly from 1 to 5 months and from 1 to 18 months, while fNIRS responses significantly increased from 5 to 18 months.

Our findings extend our previous investigations examining habituation and novelty responses within each modality. For the EEG paradigm, we have previously reported that infants’ novelty responses increase between 1- and 5-months of age (Katus et al., 2020). Comparing the BRIGHT projects’ UK and Gambian cohort, we observed a less pronounced increase in this developmental shift towards a novelty response in the Gambian, compared to the UK infants between 1- and 5 months of age. In line with past literature (Otte et al., 2013; van den Heuvel et al., 2015; Kushnerenko et al., 2007), 1-month-old infants showed a large ERP P3 component in response to *Infrequent*, white noise sounds compared to both other stimulus conditions. This response has been described as a primarily intensity-driven, rather than a genuinely novelty-based response (Kushnerenko et al., 2013). Prior literature has shown that from 2 to 4 months of age, a robust novelty-based response emerges, as indicated by a large ERP P3 to *Trial Unique*, novel sounds (Otte et al., 2013; van den Heuvel et al., 2015; Kushnerenko et al., 2007). As discussed in Katus et al., 2020, at the group level this novelty-based response was not seen at the 5-month age point in the Gambian cohort assessed here. Interestingly, at the 18-month age point, infants in this group do show a larger ERP P3 to *Trial Unique* compared to *Frequent* and *Infrequent* sounds. This may indicate that the development of a robust novelty response occurs on a more prolonged developmental time scale in this cohort, compared to what has been reported in prior literature. However, the inclusion of an additional age point in the present study showed that by 18 months of age, infants in the Gambian cohort do show a robust increased neuronal response to novel stimuli.

Our previous work also compared fNIRS habituation and novelty detection in the Gambian compared to the UK BRIGHT cohorts at 5 and 8 months of age (Lloyd-Fox et al., 2019). We found that in contrast to the UK cohort, infants in the Gambian cohort did not show evidence for novelty detection at either of these age points, but rather showed a continued pattern of response decrements across both familiarisation and novelty trials. However, inclusion of the 18-month age point in the current study showed significant developmental gains in novelty detection between the 5 and the 18 month age points as well as more rapid habituation within the familiarisation phase at 18 months of age, which in contrast to 5 and 8 months occurred within the first 10 stimulus repetitions.

### 4.2 Cross-modal correlations of habituation and novelty detection indices

We further examined correlations between indices of habituation and novelty detection across the two assessment modalities. Such analyses are not usually feasible in neurodevelopmental research: reliance on high-quality neuroimaging data of infants across two assessment modalities and several age points requires large sample sizes in order to be able to draw meaningful conclusions. For this reason, a cohort comparison between our Gambian and UK cohort was not conducted as part of the current study. We found several positive correlations for both habituation and novelty detection across the three age points: correlations for habituation were found at the 1- and 5-month age point, whereas novelty detection responses only showed a significant correlation at the 5- and 18-month age point. Across domains, we found consistent correlations at the 5 months age point. This could indicate that even though not yet apparent at a group level, individual differences in habituation and novelty detection are more representative of the rapid underlying neurodevelopmental change accompanying this period. Once established, neural metrics of these processes might not capture individual developmental patterns as consistently, leading to notable group-level differences, but less meaningful individual differences. Previous research has suggested that the first months of life could be critical for the development of the fundamental processes we studied here (Otte et al., 2013; van den Heuvel et al., 2015; Kushnerenko et al., 2013), therefore warranting increased attention 1) in the context of association with risk and environmental factors, and 2) in terms of its predictive validity for later neurodevelopmental outcomes (Katus et al., 2022).

Despite the consistent cross-modal correlations, a substantial amount of variance remains unexplained. While this is likely to be partially driven by measurement noise in each modality, we also need to consider what unique aspects of habituation and novelty detection may be captured by each measure. While the EEG measure provides insight into basic sensory processes, the fNIRS paradigm examines a much more subtle process, namely a change in a speaker's sex. Given the different levels at which these two processes operate, it is interesting to see that the two measures do share some overlap. While sensory discrimination as measured by the EEG paradigm undoubtedly represents an important building block for detecting the speaker change used in the NIRS paradigm, other factors, such as infants’ early social interactions and exposure to infant-directed speech come into play when detecting a speaker change. One reason this association may have become apparent between the very different paradigms, lies in the specific ERP indices we extracted: by examining the P3, which indexes selective attention, information processing and working memory updating, we may have tapped higher order cognitive processes, which were more similar to the underlying processes required during the fNIRS paradigm.

Our current results do not support the assumption that across the first 18 months of infancy there is a shift in the underlying neural structures supporting habituation and novelty detection. While both fNIRS and EEG showed functional changes, with a robust novelty response emerging at around 18 months of age, we did not find evidence on the basis of the fNIRS paradigm that cortical areas associated with this functional change were localised to different regions at 18 compared to 1- and 5 months of age. While some primate evidence suggest that the involvement of the frontal lobes might increase with age, it might be that this shift occurs later on in humans, who are known to have a very protracted time course for frontal lobe maturation.

### 4.3 Robustness of individual responses in habituation and novelty detection

Our study also explored whether the robustness of infants’ novelty responses was associated with their habituation patterns on an individual level. We found that this was indeed the case, with a larger proportion of the infants showing habituation responses also showing novelty responses. The congruence was similar across all age points with around 90% of infants who showed a robust novelty response in both EEG and fNIRS also showing a habituation response in at least one of the modalities. This finding bears special relevance, as it highlights how common developmental trends can be captured by two vastly different measures: not only do fNIRS and EEG measure different underlying neural processes, but also the different paradigm set ups used in both modalities assess the underlying neurocognitive processes in different ways. Whereas the EEG paradigm measured habituation to simple, auditory input presented with intermittent interruptions of other sounds, the fNIRS paradigm presented complex verbal input with a relatively subtle speaker change. Our data however suggest that despite the differences in paradigm design, similar developmental trends can be measured. It also shows, that on an individual level, there is a correlation between habituation and novelty detection processes across development.

### 4.4 Limitations and future directions

Results from this study need to be regarded in the context of some limitations. First, while we do present positive correlations between fNIRS and EEG for several age points, the magnitude of these associations is small. This may be in part driven by the multiple differences in study design and stimulus type. The timescales of EEG and fNIRS responses (i.e., rapid neural response versus slower haemodynamic response) necessitate different approaches with regard to stimulus presentation, however the difference in the kind of auditory input presented in our respective paradigms (e.g., basic sensory auditory discrimination in EEG, higher level speech sound discrimination in fNIRS) may have contributed to the small size of the correlations. In this context, it is also important two note that correlations were found for two specific assessment modalities, and two specific paradigms, and further research will be required to assess if these findings apply more broadly. Furthermore, the auditory discrimination measured in our EEG paradigm may emerge earlier than the more subtle speech sound discrimination measured in our fNIRS paradigm, leading to weaker correlations in the derived neural metrics. Secondly, the need for infants to complete both the EEG and the fNIRS assessments in order to enter analyses may lead to a biased sample, where more vulnerable infants unable to tolerate headgear or long recording periods are missed. While this possibility cannot be ruled out, infants included and excluded in analyses did not differ with regard to their anthropometric indicators, sex or age. Having demonstrated that there is some correspondence between neurodevelopmental metrics across EEG and fNIRS, this might enable a higher degree of confidence in unimodal investigations in the future. In this context, it is also important to note that with a simultaneous recording of both EEG and fNIRS, data retention might have been higher as such an approach would only require the application of one headgear, and potentially a shorter administration time. However, in addition to the paradigm optimisation differences for EEG and fNIRS outlined above, as the hardware to support parallel EEG-fNIRS recordings is still being developed. At the time this project began collecting data in 2016 we were therefore confined to recording fNIRS and EEG separately. It also needs to be noted that EEG and fNIRS may be regarded as complementary measures, that differ in key domains such as 1) the underlying physiological processes of brain functioning that they measure, 2) the requirements they pose to stimulus design and presentation, and 3) coverage and location of sensors required to obtain meaningful data. Therefore, while parallel recordings have benefits, each method might lend itself more readily to specific research questions.

Further, a limitation was that for both the fNIRS and the EEG studies infants were assessed asleep at the 1-month age point, while they were tested awake in both other ages. While we have partially addressed this issue in the context of the EEG studies, by comparing subsets of infants tested asleep at 5 months to a random subset of the same size of infants tested awake (see Katus et al., 2020), we cannot fully rule out that the state change from 1- to 5 months also affected the age-related changes we observed. While we wish to investigate this issue further in the future, a core limitation within the field of research is that data on awake newborns is extremely limited, and difficult to collect. However, as the focus of the present study was in comparing responses across modalities, and state was kept constant within age points, conclusions about the cross-modal correlations can still be drawn. Lastly, we need to note that the infants in the West Kiang region in The Gambia are not routinely offered hearing screenings, which in context of auditory studies needs to be considered as a potential source of bias arising from undetected hearing impairments. While neonatal hearing screening is not part of the standard postnatal care in West Kiang, we drew on data from two auditory and social orientation items from the Neonatal Behavioural Assessment Scale (NBAS, Brazelton and Nugent, 1995), which was administered when children were 7–14 days of age. All 152 infants who were administered the NBAS showed a response to at least one of these items. In absence of clinical auditory assessments, these data provide some indication that close to birth infants showed responses on a behavioural level to auditory stimuli.

Our findings provide the basis for a number of follow-up investigations. First, we have highlighted that responses at the 5 month age point seem to be holding some significance in terms of understanding current developmental changes. It would therefore be of interest to investigate infants’ neural response patterns across both modalities in the context of environmental risk factors. Secondly, we observed slightly different developmental profiles across EEG and fNIRS, with habituation responses being apparent from the 1-month age point onwards in the EEG, but only becoming fully apparent in the fNIRS paradigm at 18 months of age. Through further investigation of fNIRS responses - within this paradigm across our other longitudinal age points, and across other paradigms (targeting social, functional connectivity and working memory indices) within the BRIGHT study - we plan to further understand the developmental trajectories of responses associated with habituation, attention and novelty. While complementary, there may be some differences between fNIRS and EEG with regard to their sensitivity and specificity in prediction and classification of long-term developmental outcomes. While the current study focussed exclusively on examining between-measure correlations, future analyses will be able to build on this work by assessing each measures utility to indicate which infants may go on to experience neurodevelopmental issues in the long term and to measure potential effects of early interventions. Lastly, it needs to be noted that environmental factors, such as access to education and resources, nutrition, and exposure to infectious diseases, may contribute to developmental differences between children in low-income countries like The Gambia compared to children growing up in high-income countries. As neurodevelopmental studies are conducted in larger samples and a wider longitudinal scope around the world, it would be beneficial to assess analyses such as the one presented in other contrasting settings as well.

## 5 Conclusion

Our study shows that both fNIRS and EEG neuroimaging modalities elucidate common features of habituation and novelty detection over the first 18 months of life. Correlations between both assessment modalities appears to be strongest for the 5-month age point, highlighting that correlations might be greatest at times of most rapid neurodevelopmental change. These findings warrant further investigations into the correlation of the development of habituation and novelty responses and environmental factors such as poverty-associated risk, specifically at the 5-month age point where robust correlations across modalities and processes were found. Additionally, an in-depth analysis of the fNIRS response patterns across additional longitudinal age points and paradigms will enable a better understanding of the developmental trajectories of these responses in the context of environmental factors within this rural Gambian population. Our findings suggest that cross-modal investigations of infants in low-resource settings, while challenging, can help advance our understanding of neurodevelopmental processes in previously understudied populations, and increase confidence of future studies in the robustness and meaning of the extracted neurodevelopmental metrics.

## Funding

The BRIGHT project is funded by the 10.13039/100000865Bill and Melinda Gates Foundation Grants OPP1061089 and OPP1127625. The Nutrition Theme at MRCG at LSHTM is supported by the MRC & the Department for International Development (DFID) under the MRC/DFID Concordat agreement (MRC Programme MC-A760-5QX00). This work was further supported by an ESRC Postdoctoral Fellowship held by Katus, grant number ES/T008644/1 and a UKRI Future Leaders Fellowship, grant number MR/S018425/1, held by Lloyd-Fox. S.E.M. and S.M. are supported by a Wellcome Trust Senior Research Fellowship 220225/Z/20/Z. This work is partly supported by the NIHR GOSH BRC. The views expressed are those of the author(s) and not necessarily those of the NHS, the NIHR or the Department of Health.

## Declaration of Competing Interest

The authors declare no conflict of interest or competing interest.

## Data Availability

Data will be made available on request.

## Appendix Supplementary materials

Image, application 1
