# Supplementary material for: Longitudinal fNIRS and EEG metrics of habituation and novelty detection are correlated in 1–18-month-old infants
Source: Neuroimage. 2023 Jul 1;274:120153. doi: 10.1016/j.neuroimage.2023.120153 (PMC10199411; doi:10.1016/j.neuroimage.2023.120153)
Supplement: Supplementary file 1 [file mmc1.docx]

# Longitudinal fNIRS and EEG metrics of habituation and novelty detection are correlated in 1-18-month-old infants

Supplementary Material


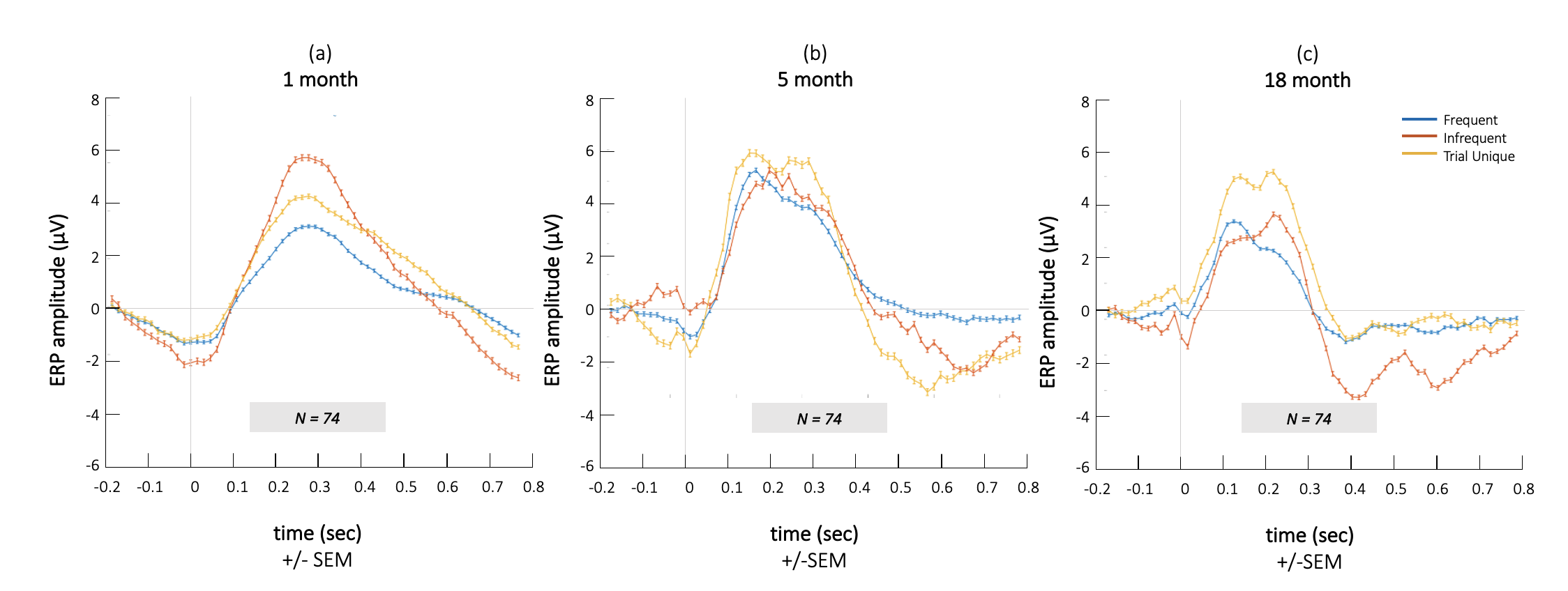


Supplementary Figure 1. ERP responses at 1-month (a), 5-months (b) and 18-months (c) of age for *Frequent* (blue), *Infrequent* (red) and *Trial Unique* (yellow) sounds. Here, only infants contributing EEG data to all three age points (N=60) are included.


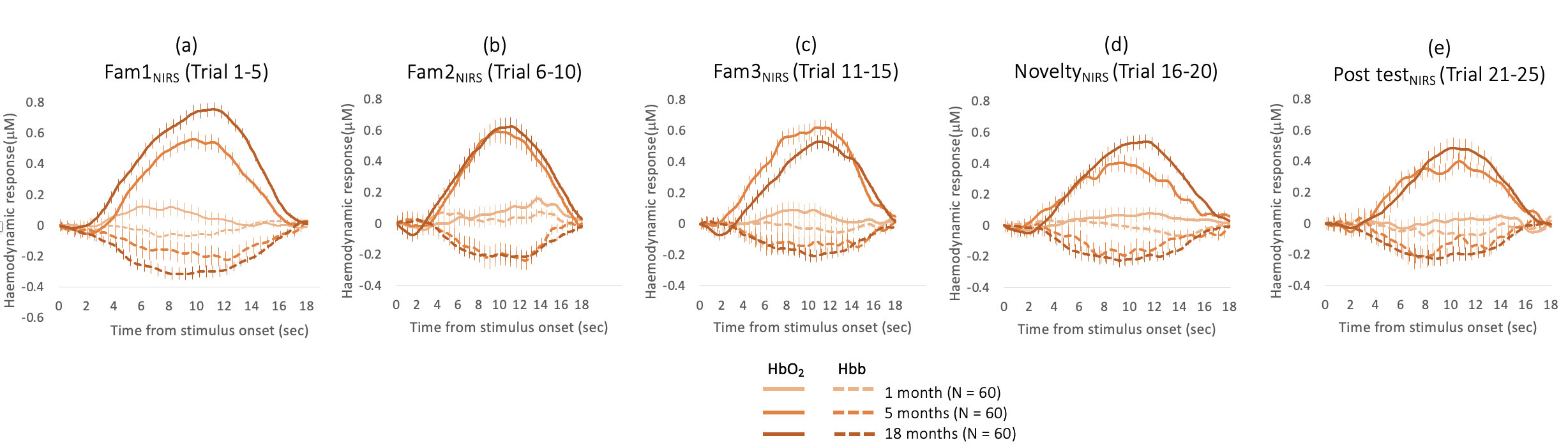


Supplementary Figure 2. fNIRS time courses at 1 month (light orange), 5 months (orange) and 18 months (dark orange) across Fam1_NIRS_ (a), Fam2_NIRS_ (b), Fam3_NIRS_ (c), Novelty (d) and Post-test (e) epochs. Here, only infants contributing fNIRS data to all three age points (N=60) are included.
